# A survey of transcriptome complexity in *Sus scrofa* using single-molecule long-read sequencing

**DOI:** 10.1093/dnares/dsy014

**Published:** 2018-05-29

**Authors:** Yao Li, Chengchi Fang, Yuhua Fu, An Hu, Cencen Li, Cheng Zou, Xinyun Li, Shuhong Zhao, Chengjun Zhang, Changchun Li

**Affiliations:** 1 Key Lab of Agriculture Animal Genetics, Breeding, and Reproduction of Ministry of Education, College of Animal Science and Technology, Huazhong Agricultural University, Wuhan, China; 2 Germplasm Bank of Wild Species, Kunming Institute of Botany, Chinese Academy of Sciences, Kunming, China

**Keywords:** single-molecule sequencing, full-length, novel gene, alternative splicing, methylation

## Abstract

Alternative splicing (AS) and fusion transcripts produce a vast expansion of transcriptomes and proteomes diversity. However, the reliability of these events and the extend of epigenetic mechanisms have not been adequately addressed due to its limitation of uncertainties about the complete structure of mRNA. Here we combined single-molecule real-time sequencing, Illumina RNA-seq and DNA methylation data to characterize the landscapes of DNA methylation on AS, fusion isoforms formation and lncRNA feature and further to unveil the transcriptome complexity of pig. Our analysis identified an unprecedented scale of high-quality full-length isoforms with over 28,127 novel isoforms from 26,881 novel genes. More than 92,000 novel AS events were detected and intron retention predominated in AS model, followed by exon skipping. Interestingly, we found that DNA methylation played an important role in generating various AS isoforms by regulating splicing sites, promoter regions and first exons. Furthermore, we identified a large of fusion transcripts and novel lncRNAs, and found that DNA methylation of the promoter and gene body could regulate lncRNA expression. Our results significantly improved existed gene models of pig and unveiled that pig AS and epigenetic modify were more complex than previously thought.

## 1. Introduction

Domestic pig (*Sus scrofa*) is an agriculturally important species and an attractive biomedical model because of its anatomical, physiological, pathological and genomic similarities to humans.^1^^,^^2^ However, increasing number of studies have shown that reference genomes are often incomplete and has annotation and structural defects.^3^^,^^4^ Thus, reference assembly and gene annotations require refinement. Obtained through short-read sequencing, the sequence data of several species have been accumulated in recent years. But the knowledge on full-length (FL) sequences of mRNAs remains scarce. Furthermore, in some cases, low-quality transcripts derived from short-read sequencing can result in incorrect annotations.^5^ FL transcripts can significantly increase the accuracy of genome annotation and transcriptome characterization. Several expressed sequence tags (ESTs) from FL studies have been performed in pigs,^6^^,^^7^ which improved genome annotation and was beneficial to downstream analysis such as expression quantification and alternative splicing (AS) identification.

AS increases the variability of the cells and tissues proteome according to different splice modes in a single animal, thereby changing the composition of transcribed genes without massively increasing the number of genes.^8^ Since AS was discovered in 1977,^9^ a large number of AS events were identified in the reference genomes of human and other animals.^10^^,^^11^ In humans, ∼20% of multi-exon genes are tissue specific and ∼95% of multi-exon genes are alternatively spliced.^10^ In porcine, ∼30% of the genes undergo AS, and 31% of the identified splice events appear to be species specific.^7^ AS is an important regulatory mechanism involved in gene expression and proteome diversity in individual^12^ and related to many diseases such as cancer and chemoresistance.^13^ Depending on a specific AS switch, enhancing the specific exon inclusion has potential as a clinically compatible therapeutic target.^14^ Accordingly, a more comprehensive and accurate identification of AS event will facilitate further cognition of the AS regulatory mechanism, scholars in medicine, genetics, bioinformatics and other fields.^15^

Diverse classes of epigenetic regulation, ranging from ncRNA to methylation, have emerged as key regulators of gene expression, genome stability and defence against foreign genetic elements.^16^ Epigenetics can mediate disease aetiology through isoform variations of cytosine modification-specific transcript which are attributable to AS.^17^ Recently, it is revealed that CHG methylation could repress AS while CG methylation promoted AS in plant.^18^ However, the comprehensive relationship between DNA methylation and AS or lncRNA remains unclear in animals. Because uncertainties about the complete structure of mRNA transcripts limited the identification of splice sites and lncRNA.

Single-molecule real-time (SMRT) sequencing carried out in Pacific Bioscience RS (PacBio, http://www.pacificbiosciences.com/) provides a third-generation sequencing platform widely used in genome sequencing because of its long reads (average: 12 kb).^19^ SMRT technology without assembling sequencing read provides direct evidence for comprehensive analysis of splice isoforms of each gene and can improve the annotation of existing gene models.^20–22^ Recently, Iso-Seq is used to analyse FL splice isoforms in humans^20^ and chickens,^22^ and indicates that identification of genes and splice isoforms are far from being complete even in a highly characterized transcriptome. In this study, we combined SMRT sequencing and short-read next-generation sequencing technology to generate a more complete FL porcine transcriptome further to analyse features of AS, fusion isoforms and lncRNAs. Accordingly, this study highlights the splice isoforms and transcriptome diversity and dynamics, provides a valuable resource for further investigation of genome annotation and increases our understanding of the porcine transcriptome.

## 2. Materials and methods

### 2.1 Animal materials

A total of 38 porcine tissues of Large White sow were collected, including 20 tissues [heart, liver, spleen, lung, kidney, stomach, duodenum, cecum, inguinal lymph nodes, precaval vein blood, ovary, uterus, corpus luteum, inner ear, subcutaneous fat, longissimus muscle, psoas muscle, soleus muscle, extensor digitorum longus (EDL) and tongue] from adult sow, and 17 tissues [heart, liver, spleen, lung, stomach, duodenum, inguinal lymph nodes, precaval vein blood, uterus, thymus, skin (dorsum), subcutaneous fat, longissimus muscle, psoas muscle, soleus muscle, EDL and tongue] from one-day-old sow and one organ (26-day-old embryo). For each tissue, total RNA was extracted using TRIzol reagent (REF15596026, Invitrogen) and processed following the manufacturer’s protocol.

### 2.2. PacBio library construction and sequencing

Equimolar rations of 38 samples were pooled together. Total RNA (1 μg) was reverse-transcribed into cDNA using the SMARTer™ PCR cDNA synthesis kit (Takara Biotechnology, Dalian, China) and optimized to prepare high-quality and FL cDNAs. Subsequently, size fractionation (0.6–1, 1–2 and >2 kb) was conducted using the BluePippin™ Size-Selection System (Sage Science, Beverly, MA). Another amplification was performed using 12–14 PCR cycles. Large-scale PCR products were purified with AMPure PB magnetic beads. Each SMRTbell library was constructed using selected cDNA (500 ng) with the Pacific Biosciences DNA Template Prep Kit 2.0. The SMRTbell templates were bound to polymerases using the DNA/Polymerase Binding Kit P6 and v2 primers. The polymerase-bound template was bound to zero-mode waveguide using Magbeadbingding kit (part 100-133-600). A total of 20 SMRT cells, composed of three SMRTbell libraries (0.6–1 kb: 7 cells; 1–2 kb: 7 cells; >2kb: 6 cells), were prepared on the Pacific Bioscience RS II platform by Frasergen Inc. (Wuhan, China) using C4 reagents with 240 min movies.

### 2.3 Illumina RNA-seq library construction

In parallel, eight tissues (subcutaneous back fat, soleus muscle, EDL and endometria from adult and one-day-old sows) from the 38 tissues were sequenced respectively using PE125 sequencing on the Illumina HiSeq 2500 platform to quantify gene/isoform expression. HiSeq library was constructed using NEB kit. Briefly, poly(A) + RNA transcript was isolated from the total RNA (1 μg). Libraries were prepared using the NEBNext Ultra RNA Library Prep Kit for Illumina (*NEB* #E7530).

### 2.4 Subread processing and error correction

**Figure 1:**
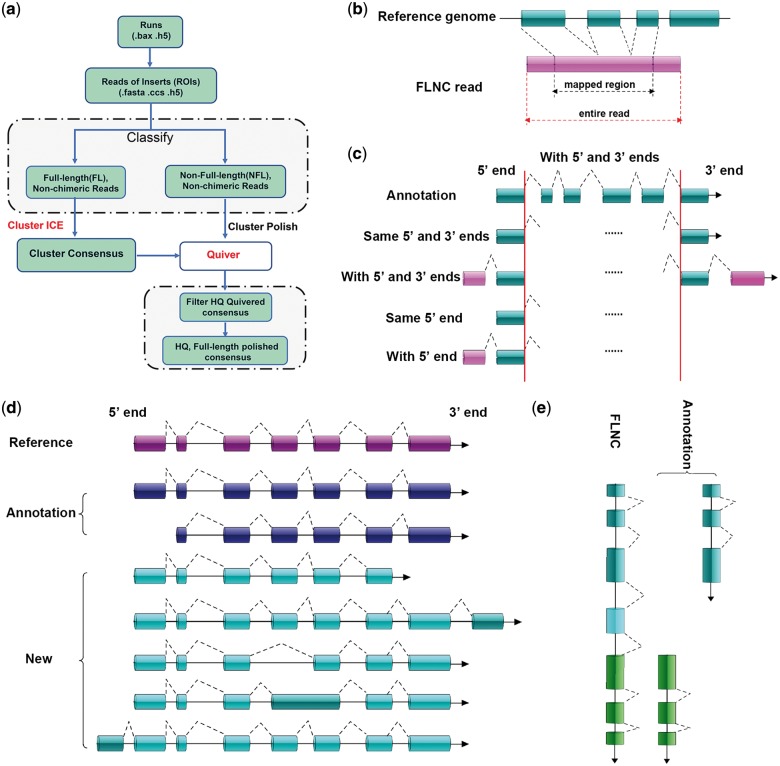
Illustration of methods. (a) Iso-Seq workflow for data processing (ToFU). (b) Mapping of PacBio data. (c) Identification of full-length. Same 5′ and 3′ ends: the first and last splice sites of FLNC were as same as the reference annotation transcripts. With 5′ and 3′ ends: the first and last splice sites of the reference transcripts were presented in the splice sites of the FLNC sequence. Same 5′ end: The first splice sites of 5′ end was identical between annotated transcript and FLNC sequence. With 5′ end: the first splice site of the 5′ end of the annotated transcript was presented in the FLNC sequence. The criteria of full-length were the type ‘with 5′ end’. (d) Criteria for assessing a new isoform. (e) Fusion transcript identification from PacBio sequences.

Effective subreads were obtained using the P_Fetch and P_Filter function (parameters: minSubReadLength =50, readScore =0.75 and minLength =50) in the SMRT Analysis Software v2.3 Suite (http://www.pacb.com/devnet/). The FL transcript sequence was obtained using ToFU pipeline^23^ (Fig. 1a). Briefly, circular consensus (CCS) read was obtained from the P_CCS module using the parameter MinFullPasses =2 and MinPredictedAccuracy =0. After examining for poly(A) signal, 5′ and 3′ adaptors, only the CCS with all three signals was considered a FL non-chimetric (FLNC) read.^24^ To improve consensus accuracy, we used an isoform-level clustering algorithm, namely, iterative clustering for error correction (ICE), and polished FL consensus sequences from ICE using Quiver with the following cut-off criteria: isoform length >200; high-quality >0.99. Additional nucleotide errors in FLNC reads were corrected using the Illumina RNA-seq data with the software Proovread^25^ using the parameter coverage of 127. The untrimmed sequence was regarded as the result of error correction.

### 2.5 Mapping of PacBio data

The error rectified FLNC reads were mapped to the pig genome sequence (Sus_scrofa.10.2.84) from ENSEMBL databases (Release 84) using GMAP^26^ with the options—no-chimeras-n 20 (Fig. 1b). The best mapped locus was selected for each FLNC read based on both identity and coverage values. Genome mapping results of FLNC reads were visualized using the Integrative Genome Viewer.^27^ The high percent of identity (PID) aligned FLNC reads were used to annotate loci and isoform. For loci, two sequences, which overlapped 20% and at least one overlapping exon to more than 20%, were identified as the same loci transcript. For isoform, single-exon sequence with overlap was determined as the same isoform. Such sequence can be identified as the same isoform when all the splicing sites of the multiple-exon sequences were identical. The redundant and false positive gene structure was removed as follows: (i) the missing 5′ end was removed; the sequence structure was a subset of other sequences (sequence structure refers to the ordered sets of all remaining cleavage sites, excluding the initiation and termination sites); the 5′ last exon spanning the intron region was determined, and the sequence was retained when it spanned the intron region; (ii) region PID <99: each transcript model kept at least two PacBio sequences; otherwise, all junctions of this sequence were annotated or supported by the junction of second-generation sequencing and (iii) the longest one was retained when the structure of two sequences was the same.

### 2.6 Novel isoform

The gene structure annotation results were compared with those of reference annotation to determine the new gene following these criteria: (1) results showed no overlap or overlapped by less than 20% of the annotated gene site, or (2) the gene overlap was more than 20%, but the gene direction was not consistent. The criteria used for a single transcript to identify novel isoform were as follows: (i) the final splice site of 3′ end changed and (ii) new intron or new exon emerged (Fig. 1d).

### 2.7 Alternative splicing classification

The relative importance of the main models of AS^28^ and the comprehensive distribution of AS structure in pig transcriptome were ascertained using Astalavista.^29^ Astalavista was also used for the classification analysis of splice type for the gene model after removing redundancy, and the simplified model constructed by IBS was visualized.^30^

### 2.8 Methylation data analysis

Methylome data from our research group (the GEO accession is GSE92417) were remapped to the pig genome assembly, and the methylation levels were calculated as previously reported.^18^ Briefly, all splice junctions from PacBio Iso-Seq transcripts were stacked (50 bp exon +50 bp intron for donor and 50 bp intron +50 bp exon for acceptor). The methylation level of each base pair was calculated as C/(C + T). The methylation of the donor site was calculated from the first nucleotide of both strands on 5′ end of the intron as C/(C + T). The methylation of the acceptor site was calculated from the last nucleotide of both strands on the 3′ end of the intron using the same formula. For lncRNA and non-lncRNA methylations, three regions were used for methylation study: 5 kb upstream transcription start site (TSS), transcript body and 5 kb downstream transcription termination site (TTS). Each region was divided into 100 bins. Each methylation ratio was calculated from the corresponding bins from all genes.

### 2.9 Fusion transcript identification

The criteria used to identify candidate fusion transcripts for a single transcript were as follows (Fig. 1e): (i) FLNC transcript mapped to two or more annotation loci in the genome; (ii) each mapped locus must align with at least 10% of the transcript; (iii) the combined alignment coverage must be at least 99%; (iv) each mapped locus must be at least 10 kb apart and (v) a certain amount of Illumina reads should support the fusion regions.

### 2.10 LncRNA identification from PacBio data

LncRNA identification was performed as previously reported.^18^ The known high-confidence 27,692 long non-coding RNA transcript sequences and 94,359 protein-coding transcript sequences of human downloaded from GENCODE (Release 25, GRCh38.p7) were used to build the model using PLEK.^31^ All PacBio isoforms were predicted on the basis of the model. The open reading frames (ORFs) of candidate lncRNAs were predicted by EMBOSS (http://emboss.bioinformatics.nl/). The transcripts encoding ORFs that were longer than 100 amino acids were filtered. The remaining transcripts were further screened by BLASTX (*e*-value ≤1*e*−10) against protein sequences of all species from NR database. Finally, Basic Local Alignment Search Tool (BLASTN) was used to eliminate the previously discovered lincRNAs of pig in ALDB^32^ under a criterion of *e*-value of ≤1e−10, min-identity of 90% and min-coverage of 85%.

### 2.11 Identification of tissue-specific and period-specific PacBio isoforms by Illumina data

Isoforms detected by PacBio was used as the template combined with the Illumina data to detect the junction of different tissue and period for multi-exon isoforms of Iso-Seq data. We used a new modified GFF file to estimate the expression level of each isoforms. The new GFF file contained junction positions of each isoform which were extracted from the GMAP mapping data. The expressed junction information was obtained as follows: the Illumina raw reads were under the quality control by ng_QC (in-house software of Frasergen Bioinformatics Inc., Wuhan, China) with parameters read_len 125 and default settings for other parameters. Then, the clean paired reads were aligned to the pig genome sequence using TopHat2.1.0^33^ following these options: G—library-type fr-first strand. When all the junctions of one isoform were supported by the Illumina data, it was defined as an expressed isoform (with ‘1’) in the corresponding period or tissue. Conversely, when no Illumina data supported the junction of the Iso-Seq data, or the junction was only partially supported by the Illumina data, it was defined as an unexpressed isoform (with ‘0’) in the corresponding period or tissue. In addition, the read counts for expressed genes were calculated with the HTSeq-count software^34^ and the differential expression level of genes were determined using GFOLD^35^ with the default parameters.

### 2.12 Validation of isoforms by RT-PCR

We randomly selected 13 isoforms including AS, novel gene, lncRNA and fusion gene for experimental validation. Transcript-specific primers were designed to span the predicted splicing events based on FL sequences using Primer-BLAST (https://www.ncbi.nlm.nih.gov/tools/primer-blast/; Supplementary Table S13). PCR amplification was monitored on 1.5% agarose gel and followed by Sanger sequencing. The 18srRNA was amplified as an endogenous control.

## 3. Results

### 3.1 Pig transcriptome by PacBio Iso-Seq

Short-read sequencing from the Illumina platform is effective in qualifying gene expression and detecting AS events. However, its capacity to accurately detect FL splice variants of genes is limited.^36^ To avoid underestimating isoform diversity, pig transcriptomes were sequenced using the PacBio Iso-Seq platform. This platform can provide long reads often up to several transcript lengths, thus the acquisition of accurately reconstructed FL splice variants is possible. To identify as many transcripts as possible, high-quality total RNA was extracted from 38 tissues at two different developmental stages. The method for detecting FLNC, new AS and fusion transcripts was depicted in the flow chart shown in Figure 1.

**Figure 2:**
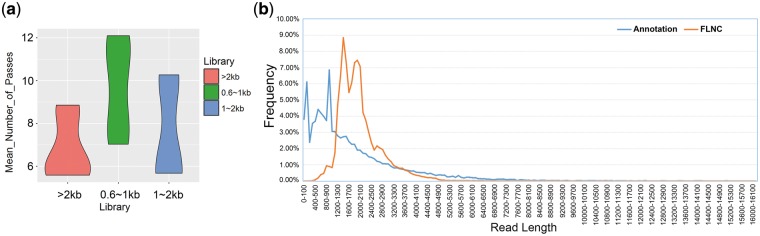
Length analysis with Iso-seq reads. (a) Mean number of passes of CCS read. (b) Comparison of length between reference annotation and FLNC.

SMRT bell libraries were constructed and sequenced on the PacBio RSII using the latest P6-C4 chemistry with 20 SMRT cells. In total, we detected 1,898,155 polymerase reads representing more than 36 G bases, with a mean length of ∼12 kb (Supplementary Table S1). After processing raw data by ToFU pipeline (Fig. 1a), we obtained 14,868,653 filtered subread data with a mean length of 2,596 bp (Supplementary Table S2) and 1,300,544 CCS reads with average depth of 6–10 passes in three libraries (median, Fig. 2a). Then CCS reads were classified into five types as follows: with 5′ adaptor, with 3′ adaptor, with poly-A tail, FL and FLNC (Fig. 1b and c). We detected 517,462 FL reads [containing 5′ and 3′ cDNA synthesis primers and a distinct poly(A) tail], of which 99.46% (514,659/517,462) were defined as FLNC (Table 1). Interestingly, we found that only 1.10% (2,254/206,756) FLNCs were <1 kb in length in 0.6–1 kb library (Supplementary Table S3), indicating that swine FL transcripts might be more than 1 kb in length while short fragments mainly consisted of non-coding RNAs with a single exon. Thus, future research on FL transcript can mainly focus on longer than 0.6 kb reads. By analysing the distribution of transcripts’ length, we found that PacBio data set could retrieve much longer transcripts than those described in the current SSC10.2 reference annotations (Fig. 2b).

**Table 1:** Sequence summary of PacBio CCS reads

Library	Cell	CCS	5'	3'	poly-A	FL	FLNC	Average FLNC read length (bp)
0.6–1 kb	7	456,861	240,085	286,237	279,184	207,721	206,756	1,560
1–2 kb	7	472,850	225,442	268,959	261,714	189,761	188,735	2,087
>2 kb	6	370,833	155,580	192,158	185,582	119,980	119,168	2,853
Total	20	1,300,544	621,107	747,354	726,480	517,462	514,659	

Considering a high base error rate of SMRT sequencing technology, we used high-quality Illumina short reads to correct erroneous SMRT long reads by Proovread software.^25^ The FLNC sequences before and after correction were respectively aligned to pig genome sequence through GMAP. After correction, we obtained 389,781 high-quality FLNC for further study (Supplementary Table S4).

### 3.2 Isoform detection and characterization

**Figure 3:**
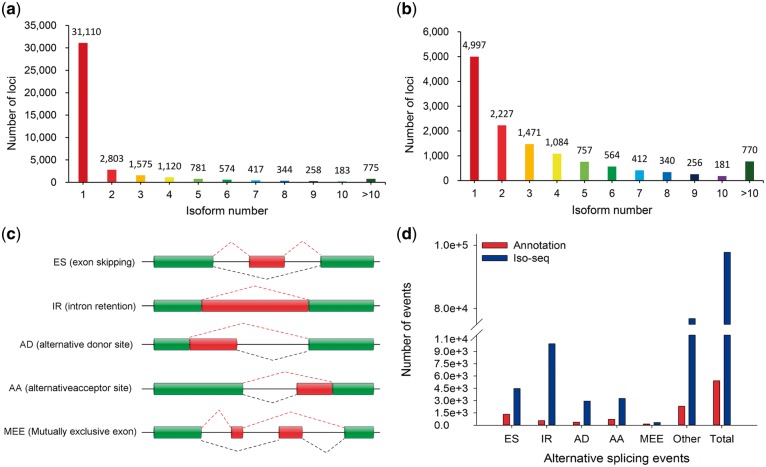
Distribution of genes that produce splice isoform and AS event with different models. (a) The total number of loci according to isoforms of FLNC in PacBio Iso-deq data. (b) Annotated loci and isoforms were based on FLNC mapped to reference genome. (c) Five basic models of AS: exon skipping, intron retention, alternative donor site, alternative acceptor site and mutually exclusive exons. (d) The total number of AS events in genes based on Iso-seq data compared with the annotated gene models.

To evaluate the density and length of isoforms, we compared the loci coverage of PacBio FLNC and swine SSC 10.2 annotation. In PacBio data set, a total of 389,781 high-quality FLNCs covered 77,075 isoforms and were allocated to 39,940 loci (Supplementary Table S5). About 96.65% (38,604/39,940) gene transcripts were ≥1 kb in length. In reference annotation, 30,585 isoforms covered 25,322 loci and only 58.72% (14,872/25,322) gene transcripts were ≥1 kb. Our unique isoforms covered about 51.57% (13,059/25,322) of reference annotation loci. In addition, out of 77,075 isoforms, 29,992 (38.91%) were single-exon isoforms and 47,083 (61.09%) were multiple-exon isoforms. Approximately 8,830 loci could produce more than one transcript, accounting for a total of 45,695 isoforms (Fig. 3a). In contrary, in reference annotation, around half (51.57%, 13,059/25,322) could be detected in Iso-Seq reads, of which only 8,062 genes (∼61.74%) could produce at least two splice isoforms (Fig. 3b). The gene *ACTA1 (*gene ID: 14.1882, chr14: 65, 236, 181-65, 239 and 183*)* had the largest number of isoforms ∼337, which played a vital role in the development of skeletal muscle myofibrils in pig.^37^ Thus, PacBio FLNC data set provided higher isoform density and longer isoform length than SSC 10.2 annotation, which would be propitious to unveil the comprehensive assessment of the true complexity of the transcriptome for the gene structure annotation.^20^

### 3.3 Novel genes identification

Compared with reference annotation, over 69% (193,318/277,842) of high-quality aligned FLNC had the same initial or terminated sites as mRNAs in annotated database, implying a relatively high integrity in structure. Considering the preferable integrity of the FLNC at the 3′ end based on the characteristics of library construction, gene structure integrity of FLNC was only estimated at the 5′ end of the sequence. After removing redundancies and false positives, a total of 237,580 FLNC (∼85.51%) contained the same initial splice site sequences with the reference annotation, covering 29,036 isoforms (∼68.34%, Supplementary Table S6). Moreover, a total of 14,792 FLNC isoforms from 6,105 genes maintained the same structures as annotation, which is similar to previous report that ∼1,4000 FL personal transcripts from complement of a pooled set of 20 human organs and tissues in a single-molecule level.^20^

The published pig genome annotation contains ∼25,322 gene models with 30,585 isoforms. In PacBio data set, 26,881 unique transcript clusters did not overlap with any annotated gene, which likely originated from novel genes (Supplementary Table S7). Even so, about 13,000 known loci (32.70%) could be mapped to 12,278 reference annotated genes (48.49% of the 25,322 gene). Thus, Iso-Seq data displayed a good example that one gene was annotated to produce a single transcript but was found to generate four splice variants (Supplementary Fig. S1a). Novel annotation of Iso-Seq could rectify the incorrect position annotation of exon of the reference (Supplementary Fig. S1b). Novel genes have emerged as a new structure and filled the blank position without annotation (Supplementary Fig. S1c).

**Figure 4:**
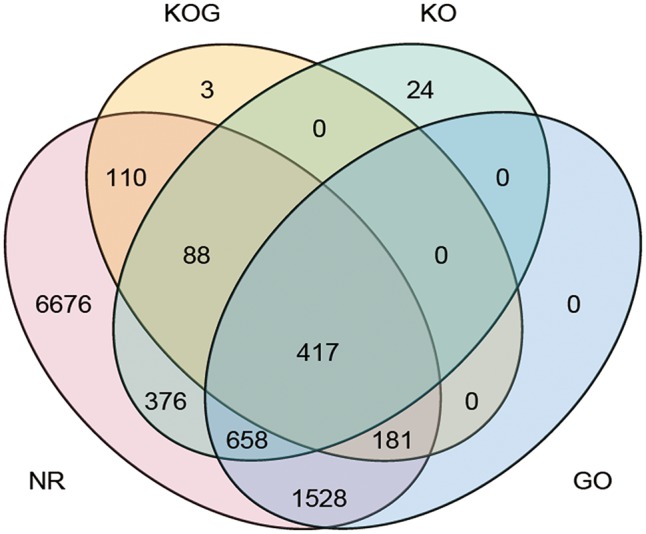
Novel genes identified in Iso-seq data. The number represents novel genes identified in NR, KOG, KO and GO databases.

Gene annotation for the 26,881 new loci showed that 23,712 loci were single-exon loci and 3,169 were multiple-exon loci (Supplementary Table S7). To validate the unannotated novel loci, we searched these 26,881 new genes in NR, KOG, KO and GO databases using BLASTX (*e*-value ≤1*e*−5). It showed that 10,299 (38.31%), 800 (2.98%), 1,567 (5.83%) and 2,803 (10.43%) of 26,881 new genes could be found in NR, KOG, KO and GO databases, respectively. A total of 417 novel genes had significant hits in the four databases (Fig. 4). At the same time, a large number of unannotated single-exon genes might contain some non-coding RNAs, because coding genes usually had multiple exons which were overwhelmingly alternatively spliced.^10^^,^^38^

### 3.4 Various types of alternative splicing

AS is an important mechanism of generating regulatory function for trait expression of eukaryotes.^39^ Through alternative recognition of exon and splice site during splicing, a single gene can generate functionally distinct mRNA and diverse protein isoforms. AS formation has five basic types as follows (Fig. 3c): exon skipping (ES), intron retention (IR), mutually exclusive exons (MEE), alternative donor site (AD) and alternative acceptor site (AA). Previously, ∼30% of porcine genes were reported to undergo AS using ESTs.^7^ In this study, we explored a more comprehensive formation and distribution of AS event in pig. In reference annotation, a total of 7.77% (1,967/25,322) genes corresponding to 17.45% (5,336/30,585) isoforms experienced 5,417 AS events (2.71 isoforms and 2.75 AS in each gene). In contrary, 17.66% loci (7,053/39,940 loci) corresponding to 42.38% (32,662/77,075) isoforms underwent 97,727 AS events in PacBio data set (4.63 isoforms and 13.86 AS in each gene, Fig. 3d, Supplementary Table S8). This result indicated that the prevalence of AS event was much higher than previously thought. Interestingly, five basic AS models only accounted for 21.42% (20,930/97,727) of AS events. IR predominated, accounting for 10.16% of alternative transcripts, while MEE (0.34%) was the least frequent. More AS events evolved from combinations of five basic patterns. For example, IR event could appear in different sites within a gene, AA event might be more prone to the 3′ terminal exon and ES event would appear in different combinations (Supplementary Fig. S1d–f).

**Figure 5:**
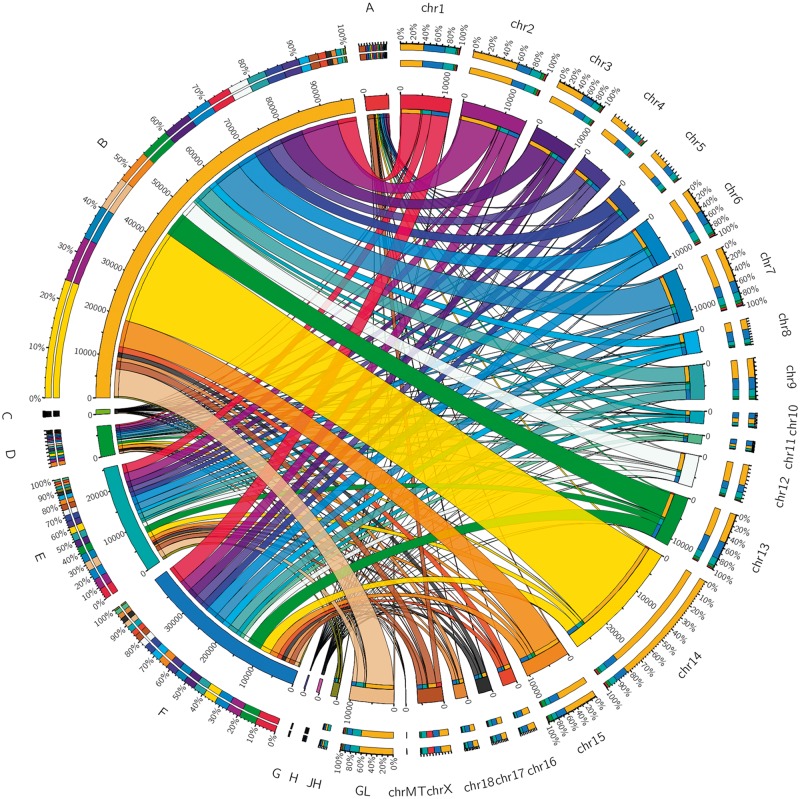
Distributed visualization using Circos of different data at chromosome level. The total length of the half circle corresponding to each label is the sum of all the values corresponding to the label. The connection between the different half circles indicates the value expressed by the two tags. A and B represent the number distribution of AS events in reference annotation and Iso-seq data, respectively. C and D correspond to number distribution of AS models in reference annotation and Iso-seq data, respectively. E and F exhibit number of gene in annotation and Iso-seq data, respectively. G and H denote fusion gene and fusion isoform in Iso-seq data, respectively. Chr represents chromosome; JH and GL represent the scaffolds that have not assembled on the chromosome.

On whole-genome level, 1,150 AS models in reference annotation and 7,132 models in Iso-Seq reads were detected (Fig. 5). In contrary, by removing the duplication among different chromosomes, 97,727 AS events with 2,637 models in PacBio Iso-Seq data were identified, of which more than 92,000 were new AS events with 2,100 modes (Fig. 3d). In detail, *GANS* gene has the largest number of splice variants (∼23 transcripts) in present reference annotation. However, we found 35 novel transcripts for *GANS* gene in Iso-Seq data, which implied a more complex imprinted expression pattern closely related to tumour therapy.^40^ Moreover, *ACTA1* gene expressed only one annotated transcript in reference but exhibited 337 splice variants in our analysis. Its specific expression in muscle provides therapeutic strategies for thin filament myopathy patients^41^ and a method for improving muscle mass in livestock.^42^ These results suggested that AS events were severely underestimated in present pig genome annotations.

Interestingly, we found that AS events (23,440 with 555 models) predominantly took place in chromosome 14. To detect whether such a situation existed in other species, we downloaded reference annotations of six species, including horse, cattle, sheep, human, mouse and pig, from ensemble (release-84, ftp://ftp.ensembl.org/pub/release-84/gtf/) for AS analysis (Supplementary Fig. S2). In other five species, correlation coefficient between the number of AS events and gene numbers was very high (*r* = 0.83–0.94). However, the correlation coefficient in pig from reference annotation was fairly low (*r* = 0.35), which was similar to the results in Iso-Seq data (*r* = 0.49). The number of AS events did not significantly grow with the increase of the number of genes on chromosome in pig, indicating that a species specificity was presented on the pig.

### 3.5 DNA methylation regulates alternative splicing variation

**Figure 6:**
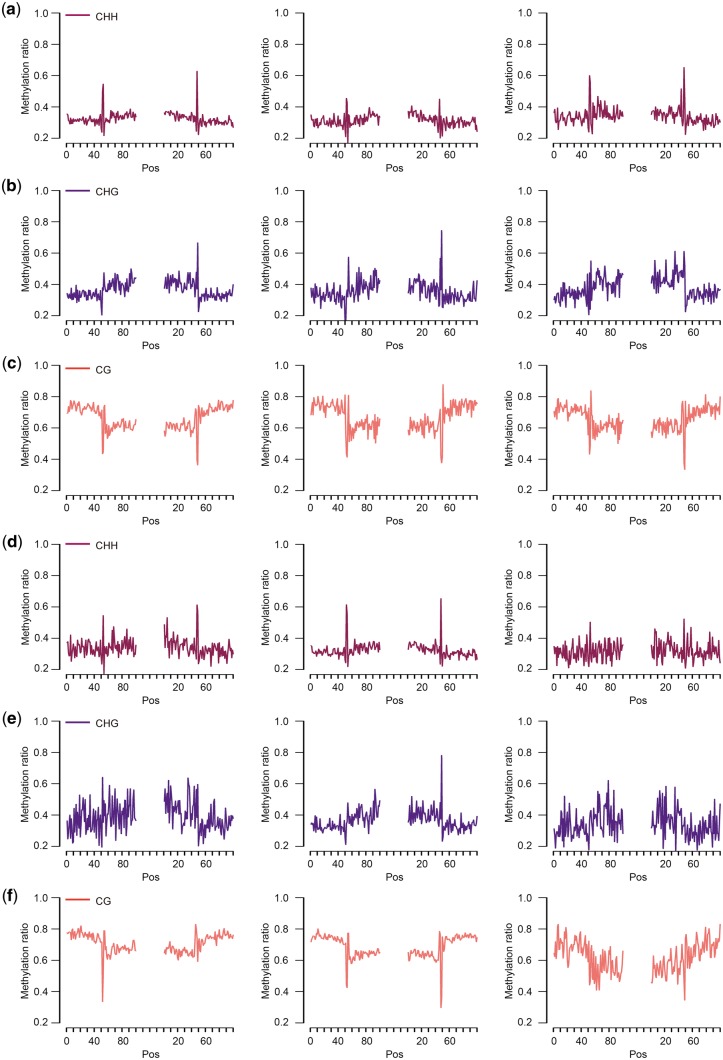
Level of DNA methylation at splice sites (exon–intron, intron–exon). (a)–(c): For each row, level of DNA methylation, combing sense and antisense strand, on the sense strand, on the antisense strand. (d)–(f): For each row, level of DNA methylation in genes with only one isoform, in all isoforms of genes with 2–10 isoforms, in all isoforms of genes with more than 10 isoforms.

**Figure 7:**
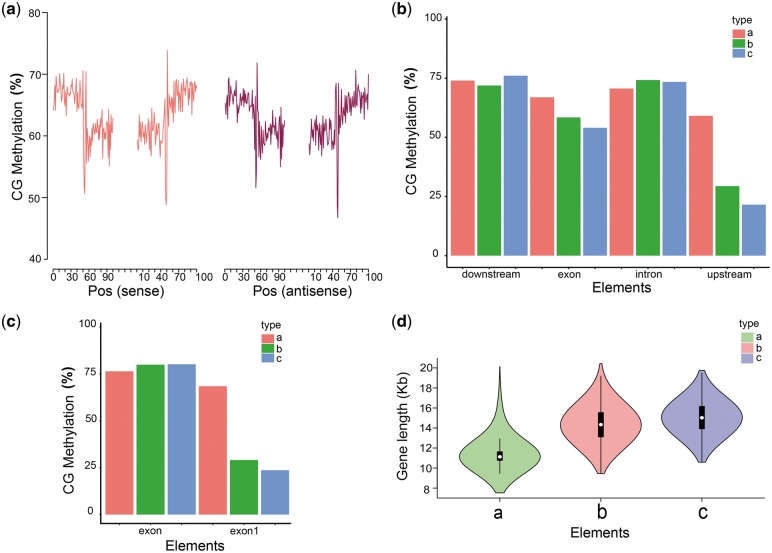
CG methylation and isoform. (a) Level of CG methylation on the sense strand and antisense strand at splice sites. (b) The relationship between methylation of gene and isoform. (c) Methylation of exon. (exon1: the first exon; exon: other exons excluding the first exon). (d) The relationship between gene length and number of isoform. In (b), (c) and (d), type a: one isoform; type b: 2–10 isoforms; type c: more than 10 isoforms.

Given that exons are often more highly methylated than introns,^43^^,^^44^ DNA methylation is often considered as a strong marker for exon–intron (EI) boundaries during splicing.^45^^,^^46^ The large number of isoforms identified via long-read sequencing in this work provided a good opportunity to investigate the relationship between DNA methylation and AS. To determine whether methylation level is related to AS, DNA methylation level of various isoforms was investigated. We stacked all splice junctions of the PacBio isoforms and measured the methylation levels of three types of cytosines on both strands in each methylation context at donor and acceptor sites. The results revealed that CHG methylation was enriched in acceptor sites, whereas CG methylation was elevated at donor sites (Fig. 6a–c). The CHH and CHG methylation levels were extremely low, whereas CG methylation predominated in pig. This result was consistent with a previous study that three types of methylated motif existed in plants, whereas more CG methylation existed in vertebrates.^47^ Simultaneously, we found that methylation levels of the longest transcripts were consistent with the methylation of all transcripts. The methylation levels at splice sites did not change significantly with the increase of the number of isoforms (Fig. 6d–f). However, we discovered that CG methylation levels dropped sharply (at least 10 times) at splice sites and then quickly increased in the range of sense strand with 3 bp and antisense strand with 2 bp [both EI and intron–exon (IE)] (Fig. 7a). The tremendous changes might be closely related to AS.

Previous research reported that the CG locus in the genome was highly methylated and the methylation of CpG islands in the promoter region of the gene could regulate gene expression.^47^ To determine whether the methylation level of gene was related to the number of isoforms, we divided the isoforms into three groups as follows: type a, genes with only one isoform; type b, genes with two to ten isoforms and type c, genes with more than 10 isoforms. Gene methylation was divided into upstream region (promoters encompass 2 kb upstream of the transcriptional start site), gene body and downstream region. Interestingly, CG methylation of promoter was negatively correlated with number of isoforms, suggesting that a high level of CG methylation at promoter region repressed AS and gene expression (Fig. 7b). Some evidence proved that the region near the promoters of many active genes tended to be unmethylated.^48^ We also found that the downward trend of methylation level of exon and promoter was insignificant when isoforms were more than 10. To determine whether the first exon was affected by CG methylation, we analysed the relationship between CG methylation of the first exon and the number of isoforms. These results showed that AS was negatively correlated with CG methylation of the first exon, suggesting that a low level of CG methylation at first exon could promote AS formations (Fig. 7c). Furthermore, we determined whether the number of isoforms was affected by the gene length. The results showed that the longer the gene, the more isoforms were produced, but the produced isoforms would be stable when the number of isoforms arrived at a certain level (Fig. 7d). The results showed that AS was not correlated with the number of genes on chromosome in pig but was closely linked to gene length and promoter methylation.

### 3.6 Fusion isoform identification and characterization

A fusion gene was an aberrant gene formed by the concatenation of two separate genes. Long-read alignments can actually determine the EI structure of fusion genes. In this study, we identified 711 isoforms and they could simultaneously cover two or more annotated genes (Supplementary Table S9). The 711 fusion isoforms corresponded to 269 FLNC fusion loci and were involved in 622 genes in the reference annotation. Distribution of fusion isoforms revealed that most of the fusion isoforms were present on chr7, followed by chr14 and chr6 (Fig. 5). In 269 FLNC fusion loci, 31 chimeric genes (∼11.52%) corresponding to 90 fusion isoforms (∼12.66%) originated from at least three candidate genes, whereas the remaining from two candidate ones. Previous studies showed that fusion events were mostly comprised of two genes.^49^ However, our results revealed that fusion event was not limited to two genes and could include more than two genes, or even ncRNAs. For example, MT.3.1 originated from 10 candidates (*MT-CO2; MT_tRNA; MT-ATP8; MT-ATP6; MT-CO3; MT-tRNA; MT-ND3; MT-tRNA; MT-ND4L; MT-ND4;*). *MT-CO2* is usually unexpressed and has the characteristic of inducible expression. Once the transcript originates from *MT-CO2* expression, it can induce tumour occurrence^50^ and increase the probability of inducing tumour in females.^51^ Moreover, fusion event could not only span two distinct genes but also produce variant isoforms with AS events. For example, fusion isoform 11.112 originated form two genes (EXOSC8-ENSSSCG00000026613) and acquired another two novel exons (Supplementary Fig. S1g). This result was consistent with previously reported results that FL fusion could produce fusion genes that acquired novel genes or exons, suggested their isoforms independent of a reference annotation library.^49^ Thus, fusion isoforms identified by Iso-Seq would provide more direct evidence for further studies of aberrant transcription which were from *trans*-splicing of distinct genes or splicing of fusion genes formed by from genome rearrangements.

To test whether these fusion events were formed stochastically by abnormal AS or necessarily by functional requirement, we analysed the functional relationship between precursor genes and each fusion isoform (Supplementary Table S9). After excluding unannotated genes, we obtained 539 fusion isoforms and 352 precursor genes. Interestingly, we found that a majority of fusion isoforms (397/539,73.65%) were formed from the combinations of separate precursor transcripts in a same gene family. For example, 58 isoforms were from different duplicates of the Myosin heavy chain gene family (*MYH*), 18 isoforms from DEAD-box helicase family (*DDX*) and 15 isoforms from interferon induced protein with tetratricopeptide repeats family (*IFIT*). Thus, fusion isoforms from a same gene family implied that the formation of fusing events were likely due to special biological functions such as functional conserved between conserved duplicates or functional complementation between subfunctional duplicates. For other fusion isoforms that originated from different family genes, precursor transcripts of which were probably functionally related. For example, a fusion gene 7.1323 originated from two precursor transcripts (*ATP6V1G2* and *DDX39B*). *DDX39B* encodes a member of the *DEAD* box family of RNA-dependent ATPases that mediates adenosine triphosphate (ATP) hydrolysis during pre-mRNA splicing.^52^ The fusion gene 7.1323 could produce 17 fusion isoforms in pigs while it only produced two transcripts in humans and might induce immunologic and infectious diseases.^53^

Moreover, we perform a more precise BLAST analysis using 711 fusion isoforms against NCBI Reference RNA Sequences database to validate fusion isoforms. Results showed that, 270 fusion genes (∼38%) are supported by at least one Reference RNA Sequences in pig (with identity >95% and overlap >90%). In addition, we searched these fusion genes in human. Interestingly, 86 are supported by at least one Reference RNA Sequences (with identity >88% and overlap >90%). And 84 fusion genes were both supported in these two databases, suggested that fusion gene may exist in different species and evolve conserved functions (Supplementary Table S9).

### 3.7 Tissue-specific and period-specific isoform

Identification of specific isoform is among the primary analysis accomplished through Illumina RNA-seq data of the same eight tissues. We used a new modified GFF file to estimate the expression level of each isoforms (Supplementary Table S10). The new GFF file contained junction positions of each isoform which were extracted from the GMAP mapping data. We obtained more than 4 G bases clean paired data each tissue, which was adequate for quantify gene expression (Supplementary Table S11). Additionally, rarefaction analysis revealed that sequencing depth had reached saturation of gene and isoform discovery in each tissue (Supplementary Fig. S3a and b). STEM^54^ expression analysis for the 39,940 loci revealed that 25,018 loci were expressed in eight tissues. Moreover, about 46.25% (12,432/26,881) of novel gene loci and 48.45% (4,282/8838) of lncRNA could be validated from these eight tissues (Supplementary Table S12).

**Figure 8:**
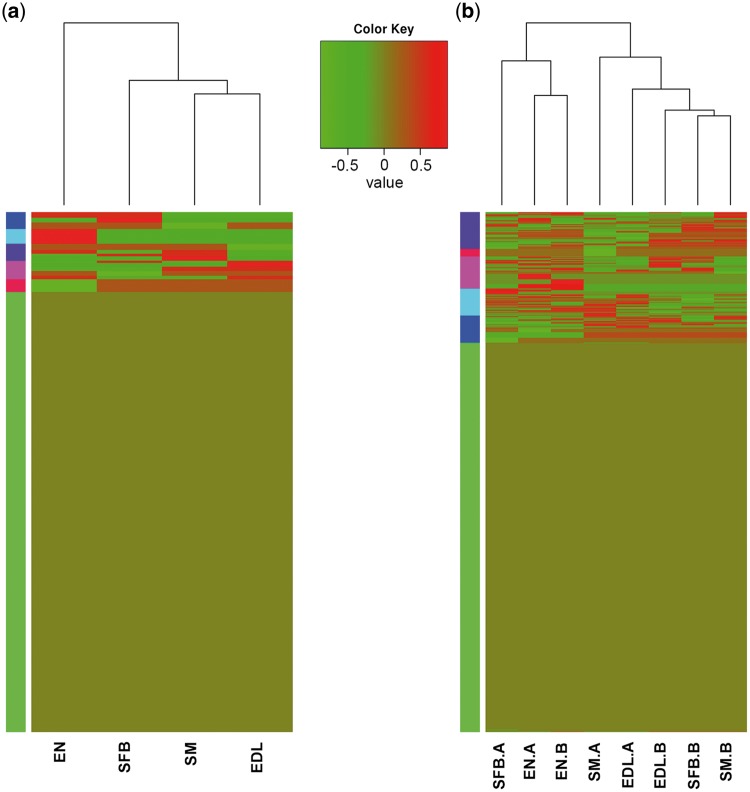
Heatmap of tissue-specific and period-specific isoforms. (a) Tissue-specific isoforms. (b) Period-specific isoforms. SFB: subcutaneous fat of back, SM: soleus muscle, EDL: extensor digitorum longus, EN: endometria, A: adult, B: birth (one day). The coloured box on the left means the cluster analysis of whole-gene expression levels in eight tissues.

**Figure 9:**
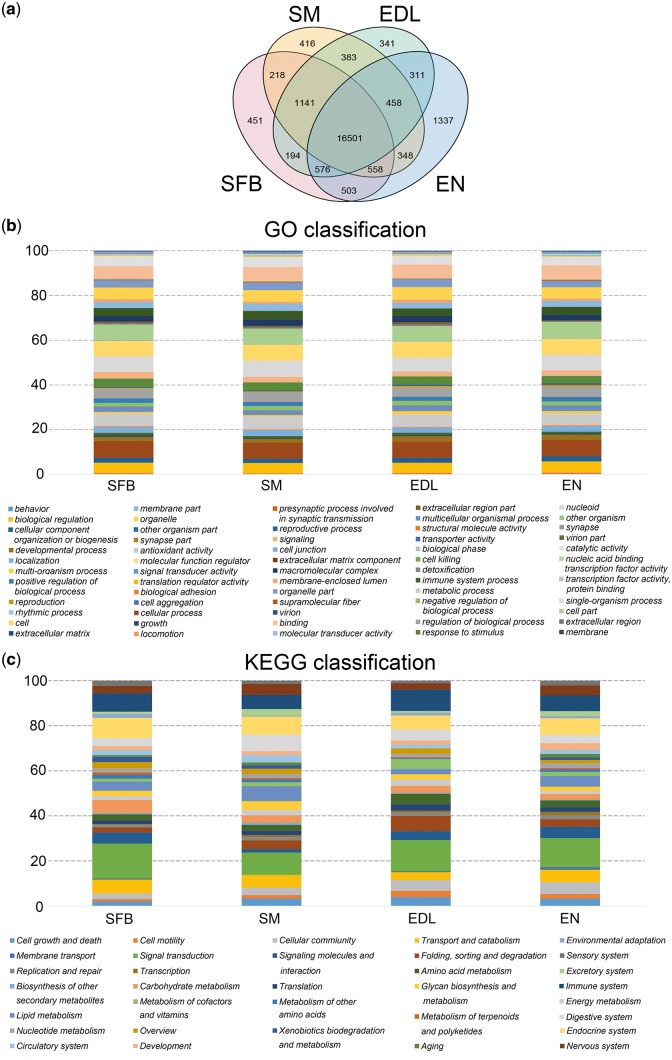
Function annotation of tissue-specific isoforms. (a) Tissue-specific isoforms. (b) Classification of gene ontology annotation for isoform uniquely expressed in the four tissues. (c) Classification of KEGG pathways annotation for isoform uniquely expressed in the four tissues.

For detecting the tissue-specific expressed isoform, a total of 47,083 multi-exon isoforms were analysed in different tissues and periods (Fig. 8). Uniquely expressed isoforms were 451 in subcutaneous fat of back (SFB), 416 in SM, 341 in EDL and 1,337 in EN respectively, and 16,501 isoforms were simultaneously expressed in the four tissues (Fig. 9a). For the 2,545 isoforms uniquely expressed in each tissue, GO terms were primarily (top six) related to cellular and single-organism processes, cell, cell part, metabolic process, binding (Fig. 9b). Function enrichments of pathway (Fig. 9c) varied slightly in each tissue such as catabolism and carbohydrate metabolism in SFB; digestive system and lipid metabolism in SM; sorting and degradation and digestive system in EDL and endocrine system, immune system and transport and catabolism in EN. These results showed that genes played the same biological functions in different tissues by the same or unique isoforms and the isoforms with tissue specificity might lead to various roles via different pathways.

For the period-specific expressed isoforms, 47,083 multi-exon isoforms were classified into five categories (Table 2). A total of 14,706 and 12,766 isoforms simultaneously expressed in four tissues in one-day-old and adult period, respectively (Supplementary Figs S4a and S5a). The number of isoforms expressed only in single tissue between one-day-old and adult also maintained high consistency (*r**** ***= 0.974). Results from GO and Kyoto Encyclopaedia of Genes and Genomes **(**KEGG) analyses of the isoforms expressed only in single tissue between one-day-old and adult revealed that the tissue diversity of the expression abundance could promote varieties of protein, although GO terms were similar (Supplementary Figs S4b and c and S5b and c). For example, isoform 12.550.1 (TUSC5) showed specific and high expression in SF adult period, which could regulate insulin-mediated adipose tissue glucose uptake by modulation of GLUT4 recycling.^55^ Isoform 3.161.1 (COX6A2), 12.685.1(MYH13) and 13.197.8 (XIRP1), with specific and high expression in SM and EDL adult period, could play important roles in skeletal muscle fibre type switch,^56^ immune-mediated myositis^57^ and muscle development,^58^ respectively.

**Table 2:** Analysis of period-specific isoforms

Isoform	1 day	Adult
Total multiple-exon isoform	47,083	47,083
Expressed in at least one tissue	22,116	21,986
Expressed in 4 tissues	14,681	12,700
Expressed only in single tissue	2,675	3,314
Unexpressed only in single tissue	2,810	2,937
Unexpressed in 4 tissues	24,967	25,097

### 3.8 LncRNA identification

**Figure 10:**
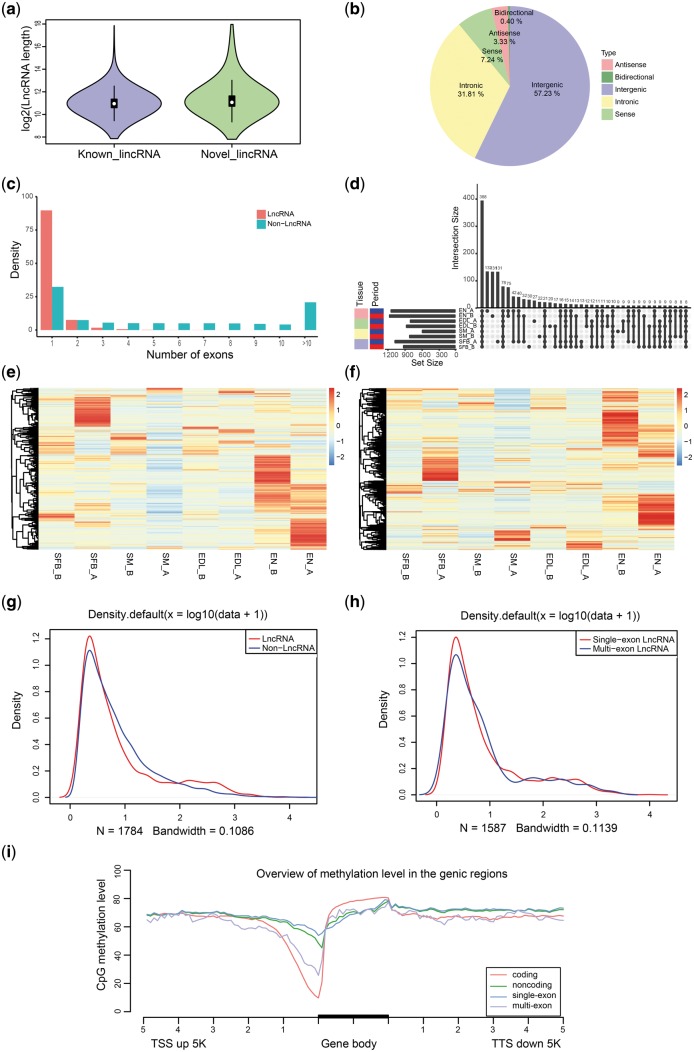
Characters of identified lncRNAs. (a) Comparison of lengths of previously reported lincRNAs with novel lincRNAs identified in our study. (b) Proportions of five kinds of lncRNAs, classified according to their position relative to protein-coding genes. (c) Number of exons in lncRNAs and non-lncRNAs. (d) Overlap of lncRNAs among tissue and period [SFB: subcutaneous fat of back, SM: soleus muscle, EDL: extensor digitorum longus, EN: endometria, A: adult, B: birth (one day)]. (e) Heatmap of lncRNA expression in eight tissues. (f) Heatmap of non-lncRNA expression in eight tissues. (g) Comparison of overall expression between lncRNAs and non-lncRNAs. (h) Comparison of overall expression between single-exon lncRNAs and multi-exon lncRNAs. (i) Comparison of DNA methylation level on lncRNAs and non-lncRNAs.

A classification model of a high-confidence set of known lncRNAs was built using PLEK^31^ to identify lncRNAs in the PacBio data. Among the 77,075 PacBio isoforms, 15,049 candidate lncRNAs were obtained via PLEK model. To obtain a high-confidence set of lncRNA genes, we eliminated transcripts with ORFs exceeding 100 codons and used BLASTX to screen the 15,049 candidates for homology with proteins of all species in NR data, thereby obtaining 8,838 lncRNAs with a mean length of 2 kb. Of the 8,838 lncRNAs, including 7,928 single-exon lncRNAs (89.7%) and 910 multi-exon lncRNAs (10.3%), 5,058 lincRNAs existed. BLASTN found that 664 of the 5,058 candidates corresponded to previously reported lincRNA of pig in ALDB.^32^ The remaining 4,394 novel lincRNAs had a mean length of 2.024 kb (Fig. 10a). LncRNAs were classified into five groups according to their biogenesis positions relative to protein-coding genes of SSC10.2.84 annotations: 57.23% of them were generated from intergenic regions, 31.81% from the intronic regions, 7.24% from the sense strand, 3.33% from the antisense strand and 0.40% belong to bidirectional lncRNA (Fig. 10b). In addition, majority (89.7%) of the lncRNAs were single exon, and this percentage was significantly higher than the non-lncRNAs (*P* < 0.01, Fig. 10c).

A total of 388 lncRNAs were simultaneously expressed in the eight tissues. Endometria at adult expressed the most specific lncRNAs (132), whereas adult soleus muscle expressed the fewest (9) (Fig. 10d). Heatmap of lncRNA and non-lncRNA expression confirmed that both lncRNA and non-lncRNA unfolded tissue-specific expression, particularly in SFB_A, EN_A and EN_B (Fig. 10e and f). Comparison of overall expression between lncRNA and non-lncRNA showed that lncRNA were significantly less expressed than non-lncRNA (*P* < 0.01, Fig. 10g). Comparison revealed that multi-exon lncRNAs expressed a higher level than single-exon lncRNAs (Fig. 10h). Furthermore, we monitored the level of CG methylation within and surrounding (5 kb upstream and 5 kb downstream) lncRNA and non-lncRNA isoforms (Fig. 10i). DNA methylation decreased near the TSS and TTS of both lncRNA and non-lncRNA genes; DNA methylation was also particularly significant for the TSS of non-lncRNA genes (*P* < 0.01). Non-lncRNA genes presented higher CG methylation within the gene body than lncRNAs, whereas lncRNAs showed higher methylation level at upstream and downstream regions than non-lncRNAs. Finally, we discovered that multi-exon lncRNAs exhibited higher CG methylation within the gene body and the lower level at upstream and downstream regions than single-exon lncRNAs. DNA methylation of the promoter and gene body was strongly correlated with lncRNA expression,^59^ which might reflect the diverse expression levels of non-lncRNA genes and lncRNAs, as well as that of single-exon and multi-exon isoforms.

### 3.9 Validation of isoforms by RT-PCR

**Figure 11:**
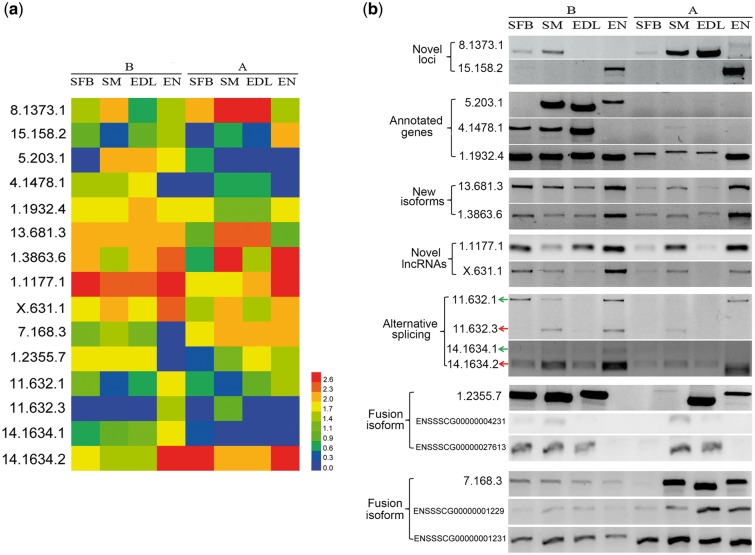
Validation of differential expressed isoforms. (a) Heatmap of differential expressed isoforms in eight tissues. (b) Validation of differential expressed isoforms by RT-PCR. The isoform symbol was explained, for example, 8.1373.1 represented the first transcript of loci 8.1373 on the chromosome 8. 8.1373.1, 14.1634.2 and 15.158.2 belonged to the transcripts of novel loci; 5.203.1, 4.1478.1 and 1.1932.4 were known isoforms of annotated genes of SSC10.2 reference; 13.681.3, 1.3863.6 and 11.632.1 were new isoforms came from the annotated genes of SSC10.2 reference; 1.1177.1 and x.631.1 were novel isoforms of novel lncRNAs; 1.2355.7 and 7.168.3 were fusion transcripts and the genes below them represented their precursor genes.

To verify the expression of novel isoforms and genes in eight tissues, we randomly selected six genes (with or without exons and with or without AS) and performed reverse transcription (RT) PCR following by Sanger sequencing (primers listed in Supplementary Table S13). As shown in Figure 11, expression and splicing were confirmed in our expression analysis. In addition, two fusion transcripts (7.168.3 and 1.2355.7) and two transcripts of novel lncRNAs (1.1171.1 and x.631.1) were randomly selected and experimentally validated. The results confirmed the authenticity of two chimeric RNA and tow lncRNAs. The validation above also showed that AS and fusion isoforms had differential expression and tissue and period specificity, thus increasing diversity of gene regulation as well as complexity of transcriptome.

## 4. Discussion

In this work, we applied PacBio sequencing technique to investigate transcripts, provided the first comprehensive view of splice variants in pig and illustrated the advantage of Iso-Seq in identifying FL splice isoforms. Following the latest methodologies in analysing PacBio transcriptome data, we obtained 389,781 high-quality FLNC reads, with 77,075 isoforms covering 39,940 loci, 97,727 AS events corresponding to 2,637 models, 711 fusion isoforms and 4,394 novel lincRNAs that were not previously annotated in pig. The new resource and transcriptional information would be of great value to improve pig genome annotation and livestock transcriptome research.

This experimental design aimed at maximizing transcript diversity and investigating comprehensive splice isoforms by broadly sampling 38 tissues and organs. Compared with previous studies, this experiment by far used the most number of tissues to comprehensively research transcriptome isoform via SMRT methodology.^20^ Twenty cells in our study might not well uncover more low-abundance isoforms, but the data depth was adequate to explore as many FL splice isoforms as possible when depending on the porcine genome size and comparing with previous similar studies of SMRT transcriptomes.^18^^,^^20^^,^^22^ In this study, only 0.6% of FLNCs was excavated in the 0–600 bp library, indicated that swine FL transcript might be more than 1 kb in length and short fragments mainly consisted of non-coding RNAs with a single exon. Thus, future research on FL transcript can mainly focus on longer than 0.6 kb reads.

AS plays important roles in regulating molecular, cellular, physiological and developmental processes/pathways in eukaryotes.^60^ However, the difficulty of identifying the combinations of splice-site usage by Illumina short reads limits gene model prediction. Previous studies show that ∼30% of genes were alternatively spliced in pig, compared with 68% in humans, 57% in mouse and 21% in bovine.^7^^,^^11^ Our analysis shows that ∼17.66% loci (7,053/39,940) are alternatively spliced, and ∼42.38% (32,662/77,075) transcripts are associated with AS in Iso-Seq data, while only 7.77% (1,967/25,322) genes corresponding to 17.45% (5,336/30,585) isoforms are in pig reference annotation. Thus, a large of AS gene identified in this study will greatly improve reference annotation. Relatively, gene proportion of AS in Iso-Seq data is low, owing to slightly high single-exon isoform with little coding capacity and thus may represent lncRNAs.^20^ Furthermore, 7,051 multi-exon genes (99.97%) are alternatively spliced in Iso-Seq data, which was consistent with previous reports on humans,^10^ confirming that almost all multi-exon genes undergo AS to increase transcriptome diversity.

Hypothetically, variations in the EI methylation dynamics may result in ES. In fact, alternative exon recognition mechanisms may have evolved in genes with equal exon-to-intron DNA methylation ratios, and this phenomenon is one of the biological explanations given for the variation in AS patterns between species.^61^^,^^62^ Interestingly, DNA methylation is enriched in AS sites and splicing regulatory motifs.^63^ We found that DNA methylation enriching in AS sites varied from acceptor to donor sites and from sense strand to antisense strand. The trend of CG methylation is not as significant like that in plant, revealing that CG methylation promoted AS.^18^ The sharp decline of cytosine coverage of EI and IE structure sites indicated that the change of cytosine content at 2–3 bp around the AS sites caused the change of methylation level and affected the occurrence of AS events. This finding provides evidence for the methylation of AS events depending on the structure of the AS sites. Moreover, our results revealed that CG methylation in promoter region repressed AS and regulated the first exon and that methylation of first exon also repressed AS in pig. Alternative first exon affected by the usage of alternative promoter was found to result in mRNA isoforms with distinct 5′ UTRs.^8^ This phenomenon indicates that promoter methylation can enhance the first exon to repress the AS events in animals.

In this study, we identified 8,838 high-confidence lncRNA, including 4,394 novel lincRNA in pig. Owing to the lack of a good database, we constructed the PLEK model using human data, which included the most complete lncRNAs. Relatively, media length of lncRNA in pig (2,024 bp) was only half of that of human (4,096 bp). ∼90% of the lncRNAs belonging to the single exon confirmed that candidate genes with few introns showed little coding capacity and thus might represent lncRNAs, and isoforms with more introns corresponded to known (mostly protein coding) genes.^20^ The expression of lncRNA is usually low, and we speculate that one reason is that lncRNAs with little exons rarely occur AS events. Consistent with previous work, we uncovered a high degree of tissue specificity among lncRNAs, a feature shared by other animals.^64^^,^^65^ In addition, we discovered that multi-exon lncRNAs expressed a higher level than single-exon lncRNAs. Methylation levels of the lncRNA genomic regions were significantly higher than that for the mRNA genes.^66^ Meanwhile, our work found that methylation levels of lncRNAs were higher than non-lncRNAs in the upstream region of TSS and downstream region of TTS, whereas non-lncRNAs with higher methylation levels existed in gene body. This phenomenon also appeared in single-exon and multi-exon lncRNAs, indicating that gene with high expression or coding capacity corresponds with low methylation level in TSS and TTS. However, high methylation level in gene body needs future research.

Overall, our study demonstrates that long-read sequencing complements short-read sequencing for cataloguing and quantifying eukaryotic transcripts. Based on FL transcripts, a large number of AS models and isoforms provide a more comprehensive foundation to explore transcriptome diversity. Our results revealed the unique species specificity of AS, the rule of fusion event, the specificity of isoform, the difference of lncRNA, the DNA methylation regulation on AS and lncRNA. These results not only significantly improve existing gene models of pig, but have revealed important rules and generated novel resource and information with positive implications for agricultural production or disease prevention.

### 4.1 Availability of data and material

The sequence data reported in this paper have been deposited in the Genome Sequence Archive (GSA; http://gsa.big.ac.cn/) of Beijing Institute of Genomics, Chinese Academy of Sciences, under accession number PRJCA000349.

## Supplementary Material

Supplementary Figure S1

Supplementary Figure S2

Supplementary Figure S3

Supplementary Figure S4

Supplementary Figure S5

Supplementary Table S1-13

Supplementary Table S7

Supplementary Table S8

Supplementary Table S9

Supplementary Table S11
