# Supplementary figures and images for: A survey of transcriptome complexity in Sus scrofa using single-molecule long-read sequencing
Source: DNA Res. 2018 May 29;25(4):421–37. doi: 10.1093/dnares/dsy014 (PMC6105124; doi:10.1093/dnares/dsy014)

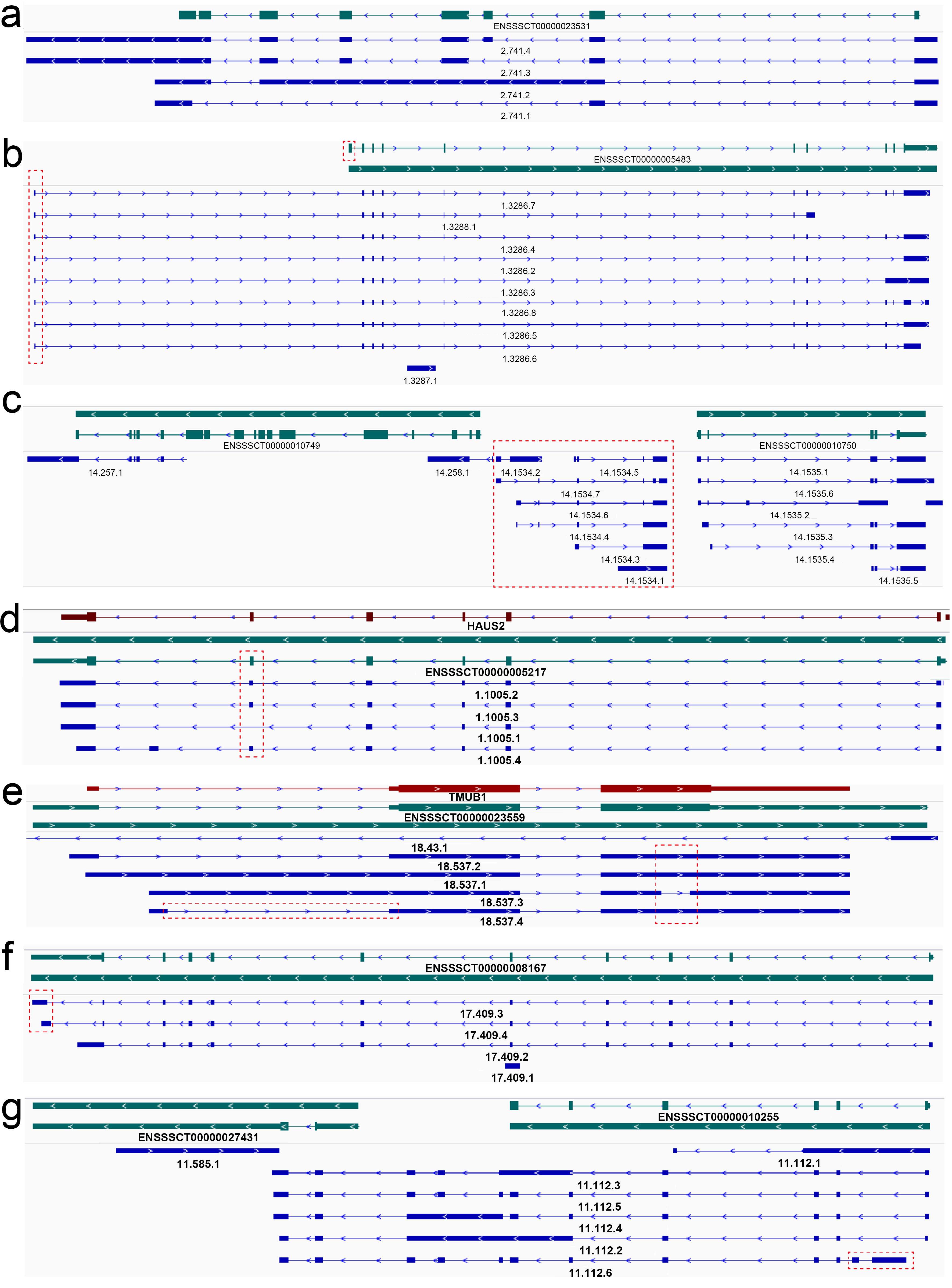

Supplement: Supplementary Figure S1 [file dsy014_figure_s1.png]

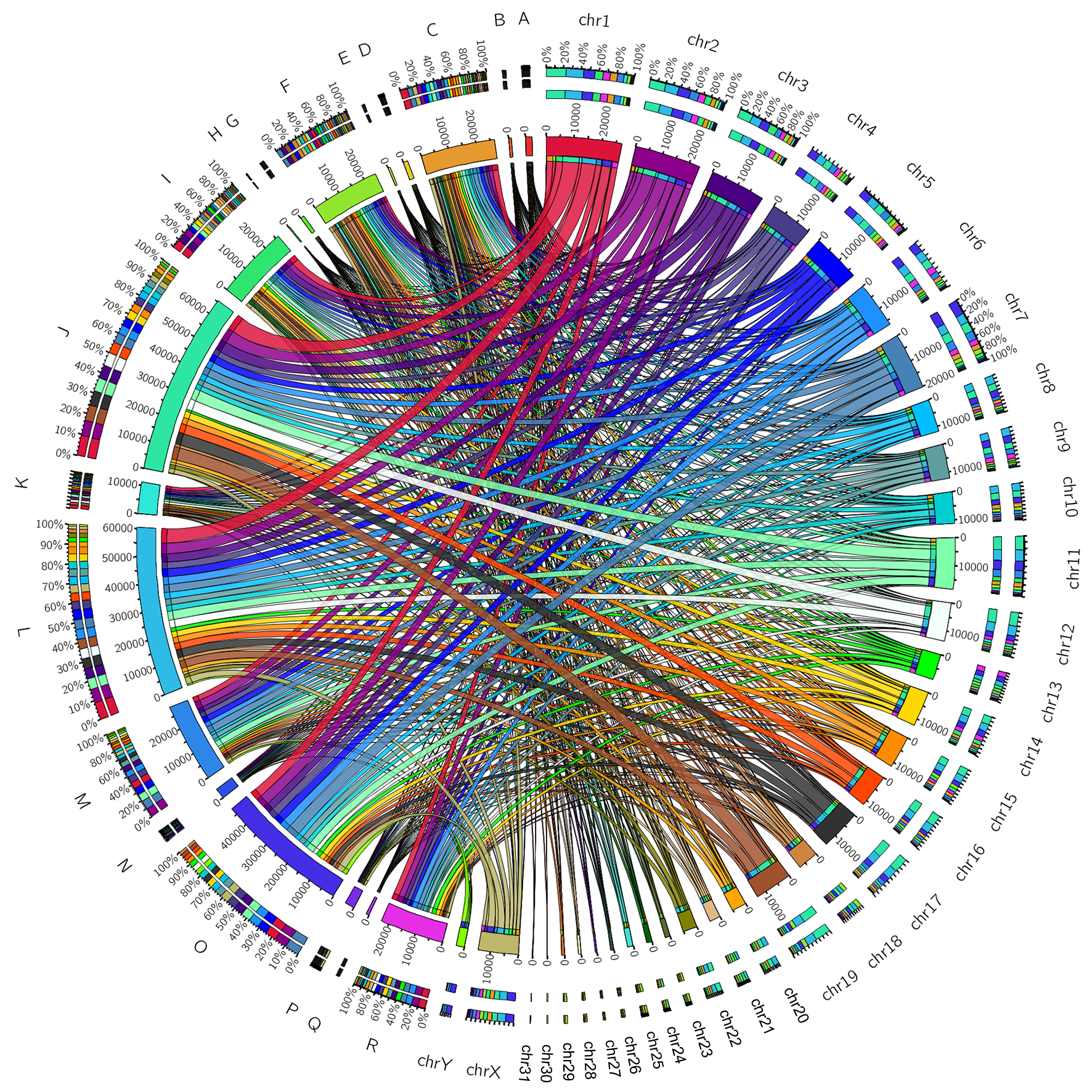

Supplement: Supplementary Figure S2 [file dsy014_figure_s2.png]

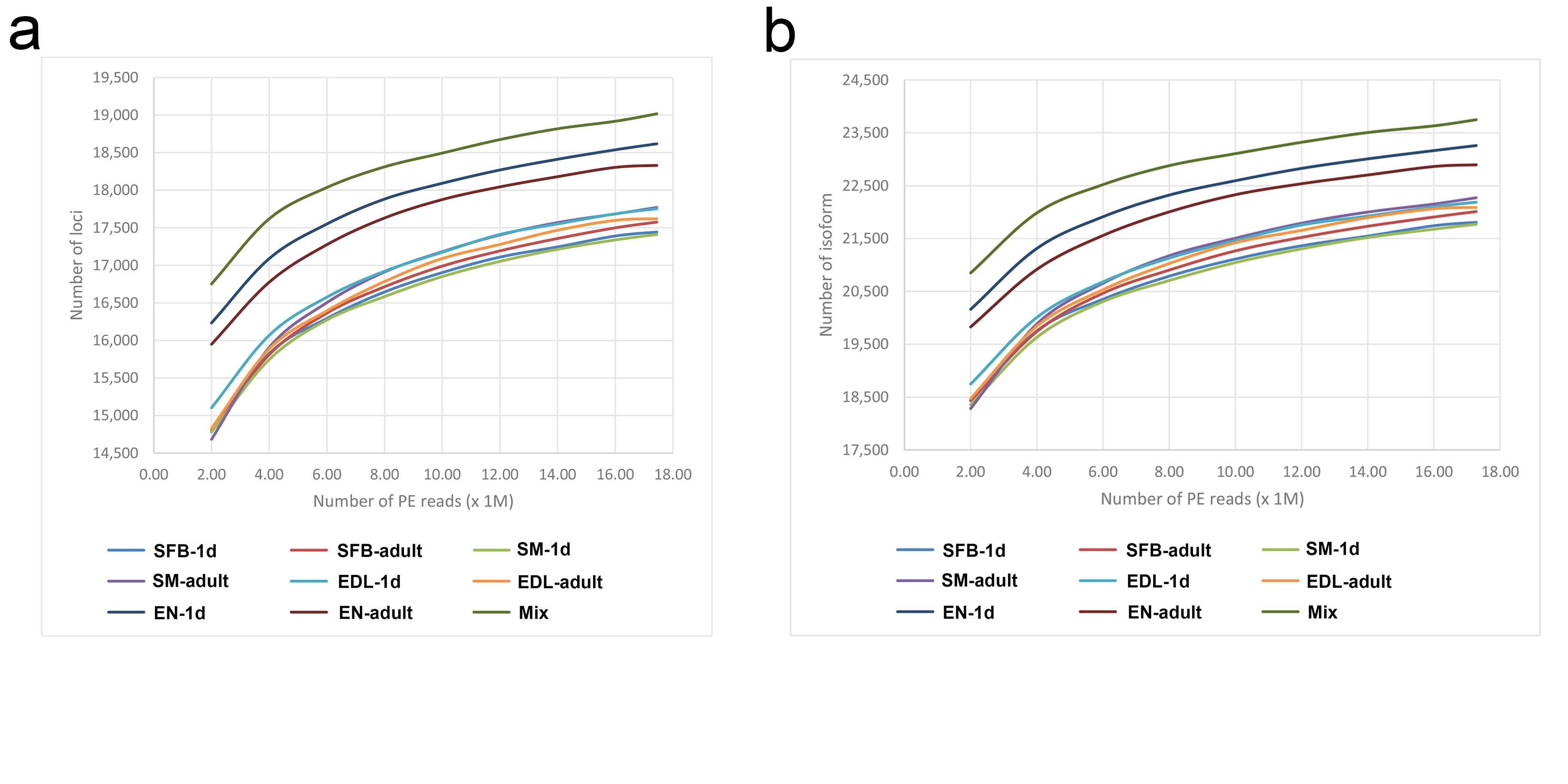

Supplement: Supplementary Figure S3 [file dsy014_figure_s3.png]

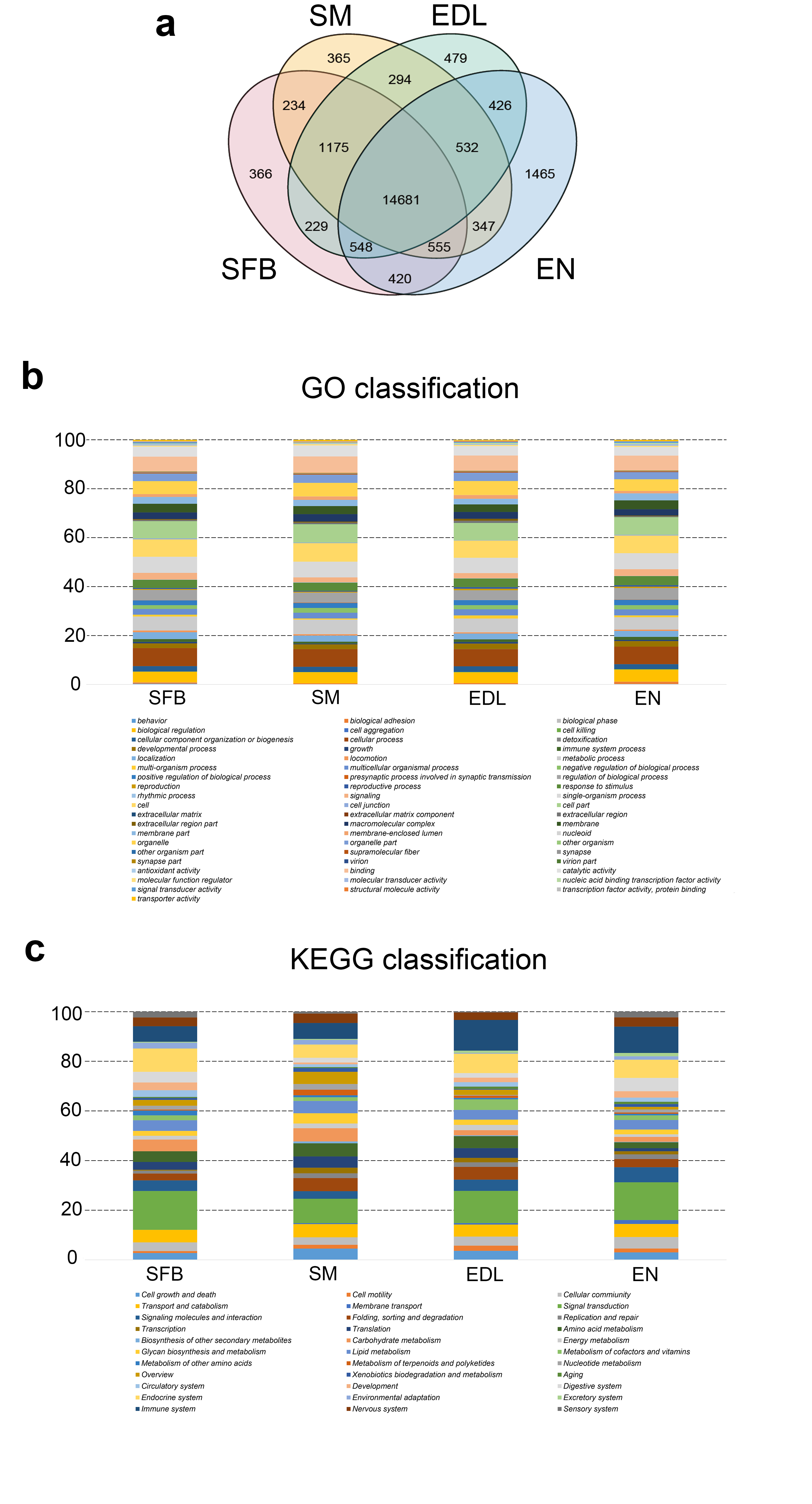

Supplement: Supplementary Figure S4 [file dsy014_figure_s4.png]

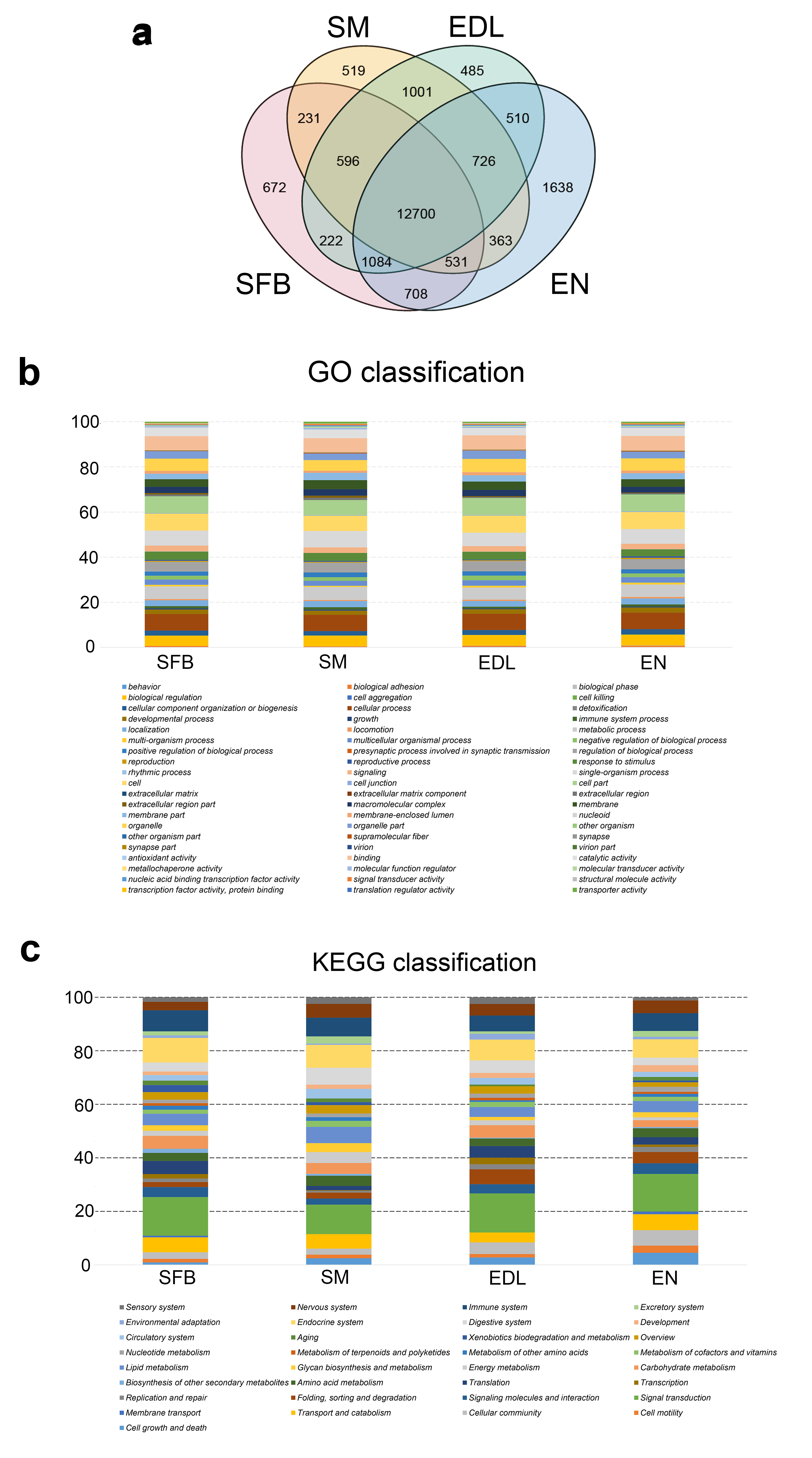

Supplement: Supplementary Figure S5 [file dsy014_figure_s5.png]
