# Supplementary material for: A survey of transcriptome complexity in Sus scrofa using single-molecule long-read sequencing
Source: DNA Res. 2018 May 29;25(4):421–37. doi: 10.1093/dnares/dsy014 (PMC6105124; doi:10.1093/dnares/dsy014)
Supplement: Supplementary Table S1-13 [file dsy014_table_s1-13.docx]

# Supporting information

**Table S1.** Sequence summary of PacBio SMRT Cells.

**Table S2.** Sequence summary of PacBio subreads.

**Table S3.** Read length <1 kb in 0.6–1 kb library

**Table S4.** Classification of FLNC sequences with genome alignment. Multiple_best align: both entire_PID and region_PID mapped to multiple site of genome. Low_PID align: entire_PID < 90%, region_PID<95%. High quality alignment: entire_PID >= 90%, region_PID >= 95%. Merge: Integrating the results of post correction and before correction

**Table S5.** Gene structure annotation.

**Table S6.** Full-length evaluation.

**Table S7.** Novel loci.

**Table S8.** Alternative splicing events of isoforms.

**Table S9.** Merged gene.

**Table S10.** Illumina RNA-seq data of each tissue.

**Table S11.** A new modified GFF file for evaluation of genes and isoforms expression.

**Table S12.** Evaluation of genes and isoforms expression by Illumina RNA-seq data.

**Table S13.** Primers designed for RT-PCR validation.

**Figure S1.** Example of alternative splicing, novel gene and fusion event. (a) Novel isoforms: an example of gene that produced 4 novel splice isoforms. The gene models contained only a single splice isoform for this gene that without FL annotation. (b) Novel annotation: the reference annotation presented incorrect position annotation on the first exon (red dashed box). (c) Novel gene: compared with the existing gene, the new gene 14.1534 showed a different structure and overlapped no annotated genes (red dashed box). (d) Exon skipping (ES): gene 1.1005 was annotated in PacBio with 4 different isoforms. A compound ES event presented between 1.1005.1 and others (red dashed box), and the 1.1005.4 was a novel isoform. (e) Intron-retention (IR): gene 18.573 was annotated in PacBio with 4 various isoforms. An IR event appeared between 18.537.3 and 18.537.4 (red dashed box). (f) Alternative acceptor site (AA): gene 17.409 was annotated in PacBio with 4 various isoforms. An AA event appeared between 17.409.3 and 17.409.4 at the last exon (red dashed box). (g) Fusion gene 11.112 spanned two distant genes and produced variant isoforms. A new exon was discovered in the annotated gene (red dashed box). Note: red: reference genome, green: reference SSC10.2 annotation, blue: PacBio annotation.

**Figure S2.** Distributed visualization of AS using Circos of different species’ annotation data at chromosome level. The total length of the half circle corresponding to each label is the sum of all the values corresponding to the label. The connection between the different half circles indicates the value expressed by the two tags. From A to R, every three tags represent AS events, AS modes and number of genes in one species, respectively, following horse, cattle, sheep, people, mouse and pig.

**Figure S3.** Rarefaction analysis of Illumina RNA-seq. (a) Rarefaction analysis of loci in Illumina RNA-seq. (b) Rarefaction analysis of isoforms in Illumina RNA-seq. SFB: subcutaneous fat of back, SM: soleus muscle, EDL: extensor digitorum longus, EN: endometria.

**Figure S4.** Analysis of Isoform specifically expressed in birth period. (a) Isoform expressed in 1 day. (b) Classification of gene ontology annotation for isoform simultaneously expressed in 1 day. (c) Classification of KEGG pathways annotation for isoform simultaneously expressed in 1 day.

**Figure S5.** Analysis of isoform specifically expressed in adult period. (a) Isoform expressed in adult. (b) Classification of gene ontology annotation for isoform simultaneously expressed in adult. (c) Classification of KEGG pathways annotation for isoform simultaneously expressed in adult.

Table S1. Sequence summary of PacBio SMRT Cells.

| Library | Cell | Pre-Filter (bp) | Post-Filter (bp) | Reads Number | Mean Length (bp) | Read Average Accuracy |
| --- | --- | --- | --- | --- | --- | --- |
| 0.6–1 kb | 7 | 12,862,987,872 | 11,966,118,767 | 651,663 | 12,226 | 0.829 |
| 1–2 kb | 7 | 12,988,995,602 | 12,389,413,696 | 676,659 | 12,346 | 0.83 |
| >2 kb | 6 | 10,237,833,356 | 10,237,833,356 | 569,833 | 11,353 | 0.827 |
| Total | 20 | 36,089,816,830 | 34,593,365,819 | 1,898,155 |  |  |

Table S2. Sequence summary of PacBio subreads.

| Library | Cell | Total Bases | NO. of Subreads | Mean Subread length (bp) | N50 (bp) |
| --- | --- | --- | --- | --- | --- |
| 0.6–1 kb | 7 | 11,551,716,250 | 6,206,983 | 1,861 | 1,852 |
| 1–2 kb | 7 | 12,047,604,247 | 5,028,072 | 2,396 | 2,516 |
| >2 kb | 6 | 9,433,351,850 | 3,633,598 | 2,596 | 3,458 |
| Total | 20 | 33,032,672,347 | 14,868,653 |  |  |

Table S3. Read length <1 kb in 0.6–1 kb library

| Reads | Total Number | Reads Number |
| --- | --- | --- |
|  |  | (Read Length <1 kb) |
| Raw reads | 651,663 | NA |
| Subreads | 6,206,983 | 799,904 (12.89%) |
| CCS | 456,861 | 136,561 (29.89%) |
| FLNC | 206,756 | 2,254 (1.10%) |

Table S4. Classification of FLNC sequences with genome alignment.

| Feature | Post Correction | Before Correction | Merge |
| --- | --- | --- | --- |
| Total FLNC | 514,659 | 514,659 | 514,659 |
| Unmapped | 14,694 (2.86%) | 18,287 (3.55%) | 14,488 (2.82%) |
| Multiple -best align | 7,878 (1.53%) | 7,645 (1.49%) | 6,584 (1.28%) |
| Low-PID align | 106,432 (20.68%) | 126,300 (24.54%) | 103,806 (20.17%) |
| High quality align | 385,655 (74.93%) | 362,427 (70.42%) | 389,781 (75.74%) |

Note. Multiple_best align: both entire_PID and region_PID mapped to multiple site of genome. Low_PID align: entire_PID < 90%, region_PID<95%. High quality align: entire_PID >= 90%, region_PID >= 95%. Merge: Integrating the results of post correction and before correction

Table S5. Gene structure annotation.

| Type | <0.6k loci number | 0.6–1k loci number | 1–2 k loci number | ≥2 k loci number | Total loci number | Single-exon loci | Multiple-exon loci | Total isoform number | Single-exon isoform | Multiple-exon isoform |
| --- | --- | --- | --- | --- | --- | --- | --- | --- | --- | --- |
| Annotation | 6,144 | 4,306 | 6,563 | 8,309 | 25,322 | 5,283 | 20,039 | 30,585 | 5,443 | 25,142 |
|  | -24.26% | -17.00% | -25.92% | -32.81% |  | -20.86% | -79.14% |  | -17.80% | -82.20% |
| PacBio | 238 | 1,098 | 17,927 | 20,677 | 39,940 | 25,487 | 14,453 | 77,075 | 29,992 | 47,083 |
|  | -0.60% | -2.75% | -44.88% | -51.77% |  | -63.81% | -36.19% |  | -38.91% | -61.09% |

Note. Annotation represents data from SSC10.2 reference annotations and PacBio from PacBio data set.

Table S6. Full length evaluation.

| Comparison with gene annotation | No. of FLNC | Ratio of full length FLNC | No. of isoform | Ratio of full length isoform | No. of Loci | Ratio of full length Loci |
| --- | --- | --- | --- | --- | --- | --- |
| FLNC mapped to multi-exon loci | 277,842 |  | 42,490 |  | 10,995 |  |
| Same 5' and 3' | 193,318 | 69.58% | 14,792 | 34.81% | 6,105 | 55.53% |
| With 5' and 3' | 216,581 | 77.95% | 21,201 | 49.90% | 7,333 | 66.69% |
| Same 5' | 219,100 | 78.86% | 24,241 | 57.05% | 7,373 | 67.06% |
| With 5' | 237,580 | 85.51% | 29,036 | 68.34% | 8,498 | 77.29% |

Table S 10. Illumina RNA-seq data of each tissue.

| **Tissue** | Clean paired reads | Clean base (bp) | Start Reads | Mapped Reads | Mapped Ratio | Unique Mapped Reads |
| --- | --- | --- | --- | --- | --- | --- |
| SFB (1d) | 16,824,303 | 4,206,075,750 | 33,648,606 | 27,353,904 | 0.8129 | 25,547,840 |
| SM (1d) | 17,236,205 | 4,309,051,250 | 34,472,410 | 28,111,080 | 0.8155 | 26,269,217 |
| EDL (1d) | 17,203,631 | 4,300,907,750 | 34,407,262 | 27,816,247 | 0.8084 | 26,062,560 |
| EN (1d) | 1,7414,686 | 4,353,671,500 | 34,829,372 | 28,265,427 | 0.8115 | 26,390,231 |
| SFB (adult) | 17,440,791 | 4,360,197,750 | 34,881,582 | 28,070,924 | 0.8047 | 26,294,309 |
| SM (adult) | 17,275,244 | 4,318,811,000 | 34,550,488 | 27,186,492 | 0.7869 | 25,507,449 |
| EDL (adult) | 16,343,758 | 4,085,939,500 | 32,687,516 | 26,833,492 | 0.8209 | 25,138,523 |
| EN (adult) | 16,471340 | 4,117,835,000 | 32,942,680 | 27,227,601 | 0.8265 | 25,412,735 |

Note. SFB: subcutaneous fat of back, SM: soleus muscle, EDL: extensor digitorum longus, EN: endometria.

Table S12. Evaluation of genes and isoforms expression by Illumina RNA-seq data.

| Type | Gene loci | Novel gene | lncRNA |
| --- | --- | --- | --- |
| Pacbio | 39,940 | 26,881 | 8,838 |
| Illumina RNA-seq support | 25,018 | 12,432 | 4,282 |
| Ration | 62.64% | 46.25% | 48.45% |

Table S13. Primers designed for RT-PCR validation.

| Isoform | Locus | Primer | Sequence (5′–3′) | Position of primer | TM（℃） | | Length（bp） | |
| --- | --- | --- | --- | --- | --- | --- | --- | --- |
| 8.1373.1 | chr8:71347078-71356254 | F | CACTCTACTTACAAACTGTTGCTGA | 1^st^ exon | 59 | 1,248 | |  |
|  |  | R | CTGGGGAGTCCTGGTCATTTT | 4^th^ exon |  |  |  |  |
| 14.1634.2 | chr14:42363119-42387102 | F | CAGCGGGACATTTCTCACCT | 1^st^ exon | 59 | 227 | |  |
|  |  | R | ATGGGCTCTTGGCTGTCTTC | 2^nd^ exon |  |  |  |  |
| 13.681.3 | chr13:76056465-76061577 | F | CTGCTCTCAGACCCCTTGTG | 1^st^ exon | 59 | 1,627 | |  |
|  |  | R | GGGGTGGGGCATGGAATTAG | 4^th^ exon |  |  |  |  |
| 5.203.1 | chr5:17250148-17272224 | F | GGCCTCCCTGGTCCTTTATG | 1^st^ exon | 59 | 811 | |  |
|  |  | R | CTCGGAAGACCGTTGGACTC | 8^th^ exon |  |  |  |  |
| 1.3863.6 | chr1:267525235-267548379 | F | AGGGACCTCATGAACAGGGA | 1^st^ exon | 59 | 1,758 | |  |
|  |  | R | CTGTGGCCCTGATATCGTCC | 10^th^ exon |  |  |  |  |
| 1.1932.4 | chr1:299754281-299758559 | F | ATCGAGTTGGCTTTCCGTGT | 1^st^ exon | 59 | 1,551 | |  |
|  |  | R | TCTTTTGTCAGGGGTCGCTC | 9^th^ exon |  |  |  |  |
| 4.1478.1 | chr4:60164015-60170179 | F | TCTTCCATCACAATGAGCAACA | 1^st^ exon | 59 | 819 | |  |
|  |  | R | TTGTGATCCTTCTCCGCTCG | 4^th^ exon |  |  |  |  |
| 11.632.1 | chr11:16802286-16804486 | F | ATGGTGAAATGAGCCCCCAG | 1^st^ exon | 59 | 2,036 | |  |
|  |  | R | AATGTATGTGCTTTTTCCCCAAA | 1^st^ exon |  |  |  |  |
| 15.158.2 | chr15:22133558-22413576 | F | TTGGTATGCCCGTGGAGTTC | 1^st^ exon | 59 | 1,556 | |  |
|  |  | R | GAAGGAGATGCCAGACCCTC | 2^nd^ exon |  |  |  |  |
| 1.1177.1 | chr1:175991082-175995571 | F | TGGTGATGTTTGTGCCCTGT | 1^st^ exon | 59 | 137 | |  |
|  |  | R | GTGGGTGGGACTGGATTGAG | 1^st^ exon |  |  |  |  |
| X.631.1 | chrX:127902429-127905102 | F | ACCAGTTAAACAAGGAAAACACA | 1^st^ exon | 59 | 2,259 | |  |
|  |  | R | CCTTCAAATAAGTTTTGTCCGTTCA | 2^nd^ exon |  |  |  |  |
| 1.2355.7 | chr1:43531360-43794933 | F | GCAGCTCTGTCCAAGCATGA | 1^st^ exon | 59 | 3,433 | |  |
|  |  | R | GCATTGCCACATCCACAAGAA | 35^th^ exon |  |  |  |  |
| 7.168.3 | chr7:24748099-24749369 | F | TGTGATCTGGAGGAAGAAGCG | 1^st^ exon | 59 | 459 | |  |
|  |  | R | TCAACTCCTCATGGCACCAA | 4^th^ exon |  |  |  |  |
